# Bio-Inspired Multi-Granularity Model for Rice Pests and Diseases Named Entity Recognition in Chinese

**DOI:** 10.3390/biomimetics10100676

**Published:** 2025-10-08

**Authors:** Zhan Tang, Xiaoyu Lu, Enli Liu, Yan Zhong, Xiaoli Peng

**Affiliations:** School of Artificial Intelligence and Big Data, Sichuan University of Arts and Science, Dazhou 635000, China; luxiaoyu@sasu.edu.cn (X.L.); lel@sasu.edu.cn (E.L.); yzhong@sasu.edu.cn (Y.Z.)

**Keywords:** rice pests and diseases, named entity recognition, natural language processing, bio-inspired approaches, deep learning

## Abstract

Rice, as one of the world’s four major staple crops, is frequently threatened by pests and diseases during its growth. With the rapid expansion of agricultural information data, the effective management and utilization of such data have become crucial for the development of agricultural informatization. Named entity recognition technology offers precise support for the early prevention and control of crop pests and diseases. However, entity recognition for rice pests and diseases faces challenges such as structural complexity and prevalent nesting issues. Inspired by biological visual mechanisms, we propose a deep learning model capable of extracting multi-granularity features. Text representations are encoded using BERT, and the model enhances its ability to capture nested boundary information through multi-granularity convolutional neural networks (CNNs). Finally, sequence modeling and labeling are performed using a bidirectional long short-term memory network (BiLSTM) combined with a conditional random field (CRF). Experimental results demonstrate that the proposed model effectively identifies entities related to rice diseases and pests, achieving an F1 score of 91.74% on a self-constructed dataset.

## 1. Introduction

Rice, one of the world’s most essential food crops, is characterized by its extensive cultivation and high productivity, playing a critical role in global food security [1,2]. However, rice production is frequently threatened by numerous pests and diseases, which not only reduce yield and quality but may also lead to significant economic losses and ecological issues [3]. Accurate and efficient identification and monitoring of these pests and diseases are crucial for enabling precision agriculture and promoting sustainable agricultural development [4,5,6].

Traditional methods for identifying rice pests and diseases primarily rely on manual observation and empirical judgment, which suffer from limitations such as low efficiency, high subjectivity, and poor scalability. In recent years, the rapid advancement of Natural Language Processing (NLP) has significantly improved Named Entity Recognition (NER), enabling the accurate extraction of structured information from unstructured agricultural texts [7]. While NER traditionally operates on textual data, its application in pest and disease management is motivated by the growing availability of digitized agricultural reports, scientific literature, and field notes that contain valuable yet underutilized information on pest occurrences, treatment practices, and spatial–temporal patterns [8]. By automatically identifying entities such as pest species, host crops, geographic locations, pesticides, and symptomatic descriptions, NER can transform fragmented textual data into actionable knowledge, thereby facilitating the development of data-driven early warning systems and supporting precise intervention strategies [9,10].

The NER task is essentially a technique based on sequence annotation. NER based on deep learning has attracted much attention due to its ability to automatically learn features [11], and some deep learning techniques are widely used in NER, such as convolutional neural networks (CNNs) and bidirectional long short-term memory (BiLSTM), Bidirectional Encoder Representations from Transformers (BERT), and a conditional random field (CRF). Zhao et al. [12] adopted a document-level attention mechanism DA-BERT-BILSTM-CRF model for agricultural NER, effectively solving the problem of label consistency of entities in different contexts. Li et al. [13] enhanced the ability to capture vocabulary boundary information based on the BERT-BiLSTM-CRF model and the strategy of multi-source information fusion. Niu et al. [14] proposed a text-enhanced BERT-BILSTM-CRF model framework for rice NER to address the challenge of lacking entity annotation data in the agricultural field. Liang et al. [15] proposed the RoBERTa-WM-BiGRU-CRF model to address the issues of uneven entity distribution and inaccurate recognition of complex terms in Chinese crop pest and disease identification and combined it with adversarial training methods to improve entity recognition performance.

The entity word formation in the Chinese agricultural field is complex and there are a lot of nested problems. For example, “entity: rice stem borer (水稻二化螟), type: pest” is composed of two entities: “entity: rice (水稻), type: crop” and “entity: stem borer (二化螟), type: pest”. Moreover, the lack of standardized dictionaries in this field makes entity recognition in agriculture more challenging. Although the aforementioned methods perform well in handling flat entities, they generally overlook the issue of nesting within entities.

To address this challenge, Deng et al. [16] drew inspiration from object detection algorithms in computer vision and proposed a method based on a multi-objective learning framework. They used deep neural networks to convert sentences into text feature maps and predicted nested entity boundaries through regression, thereby achieving effective recognition of nested named entities in text. Tang et al. [17] proposed an NER method based on a BERT encoder and a dual affine decoder to address the issues of poor robustness caused by overconfidence and limited word embedding representation ability. This method utilized pre-trained weights constructed on a large-scale biomedical corpus to enhance the representation capability of the embedding layer and utilized a dual affine decoder to represent nested entities. Additionally, a smoothing strategy was applied to improve the selection of entity boundaries by the dual affine decoder, thereby enhancing the accuracy of entity recognition. Yan et al. [18] proposed a Chinese entity recognition method that integrates entity nesting rules to address the challenges of nested entity recognition in Chinese medical texts. The method transforms the entity recognition task into a joint training task of entity boundary recognition and boundary head–tail relationship recognition and combines entity nesting rules in the decoding process to achieve accurate parsing of complex nested structures. These methods provide highly targeted solutions when dealing with nested entities, effectively driving technological progress in this field. Ke et al. [19] integrated multiple features such as pinyin and glyph, and combined them with a dual affine mechanism, specifically designed to solve the nesting problem of Chinese named entities.

CNNs are capable of extracting features from varying receptive fields, effectively capturing local semantic dependencies in text through convolutional kernels—such as n-gram features—and are widely employed in natural language processing tasks including text classification, sentiment analysis, and machine translation [20,21,22]. By utilizing multi-layer structures to progressively abstract semantic representations, CNNs effectively model hierarchical linguistic patterns spanning words, phrases, and sentences. In comparison to recurrent neural networks, CNNs exhibit superior parallel computing capability and higher feature extraction efficiency, demonstrating particular strength in short-text processing.

To address the challenge of recognizing nested entities in texts related to crop diseases and pests, this study proposes a deep learning model inspired by biological vision systems [23], which mimics the ability to process multi-scale information at the same hierarchical level. Specifically, the model employs parallel CNNs with convolution kernels of different sizes within the same layer to capture multi-granularity features, thereby improving the representation of nested structures.

The main contributions of this work are as follows:

(1) A high-quality, large-scale, and finely annotated named entity recognition dataset for rice pests and diseases was constructed through rigorous screening, deduplication, and noise reduction from authoritative data sources. The content was further validated under the guidance of domain experts.
(2) A hierarchical NER model that integrates multi-granularity feature extraction and contextual awareness was proposed. By incorporating parallel multi-scale convolutional kernels, the model significantly enhances nested boundary detection, providing an effective solution for recognizing complex entities in the domain of rice pests and diseases.

## 2. Dataset Construction

To construct a high-quality corpus, textual materials were sourced from authoritative databases, including the China National Knowledge Infrastructure (CNKI), Wanfang Data, and Baidu Baike. Relevant abstracts and semi-structured data were retrieved using keywords such as “rice + pests and diseases”. Under the supervision of domain experts, the collected data underwent rigorous screening to eliminate irrelevant content. To ensure diversity, additional relevant texts were obtained from multiple platforms such as the China Agricultural Technology Extension Information Platform and the China Pesticide Information Network. Domain experts were further invited to verify the relevance and accuracy of the samples. Following preprocessing steps including deduplication, removal of HTML tags, and standardization of punctuation, a total of 15,000 sentences were retained. To ensure annotation quality, all sentences were independently annotated by two annotators, with discrepancies adjudicated by a domain expert. The inter-annotator agreement reached a Kappa score of 0.92, indicating high consistency. The annotated dataset was then randomly split into training, validation, and test sets, with a ratio of 8:1:1.

**Figure 1:**
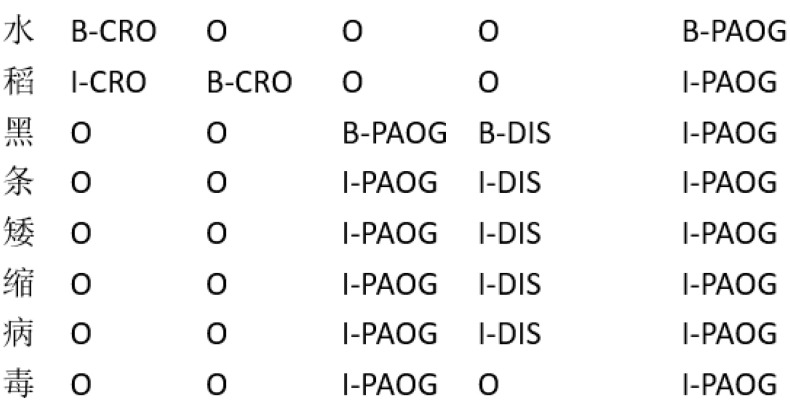
Layered tagging method (The Chinese in the figure is the original text, which menas “rice black-streaked dwarf virus”).

Currently, most general-domain datasets primarily annotate common entity types such as persons, locations, and organizations, whereas vertical-domain datasets focus on entity types within specific fields. Given the significant variations across domains, these established categories are unsuitable for identifying entities related to rice pests and diseases. In light of the lack of a unified standard for entity-type classification, we adopted the taxonomy proposed by Yao et al. [24], which categorizes agricultural pest and disease entities into 13 fine-grained types: crops, diseases, pests, drugs, fertilizers, pathogens, periods, parts, cultivars, biosystems, companies, organizations, and others. The nested named entities are annotated using the layered tagging method, and the annotation example is shown in Figure 1.

Notably, distinctions were explicitly defined during annotation, for instance, ‘Pathogens’ refers to pathogenic microorganisms such as fungi and bacteria, while ‘Pests’ denotes insect pests. The annotated dataset contains 56,657 entities (counted as flat entities), of which 35,257 contain one or more nested relationships, accounting for 62.23%. A detailed breakdown is provided in Table 1.

**Figure 2:**
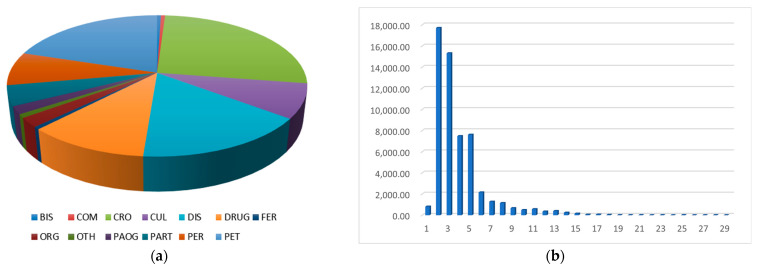
(**a**) Proportion of various entities. (**b**) Number of entities with different text lengths.

The proportion of various entities and number of entities with different text lengths are presented in Figure 2.

As shown in Figure 2a, entities belonging to Crop, Disease, and Pests account for the largest proportions, indicating that the constructed dataset is highly representative of the core concepts in the domain of rice diseases and pests.

Figure 2b illustrates that entities with text lengths between two and five characters are the most frequent, suggesting that short-span entities dominate the dataset. This implies that n-gram features within this length range (e.g., 2- to 5 g) may play an important role in text feature construction and model design for named entity recognition in this domain.

Together, these observations confirm that the dataset is well-suited for NER tasks related to rice diseases and pests, with a particular emphasis on short and medium-length entity mentions. Furthermore, the prevalence of short entities (2–5 characters in length) suggests that local contextual features within this span are particularly important for NER model design. Our data and code will be available at https://github.com/JamesTurntz/Multi-GranularityNER (accessed on 7 October 2025).

## 3. Method

### 3.1. Overall Architecture of the Model

**Figure 3:**
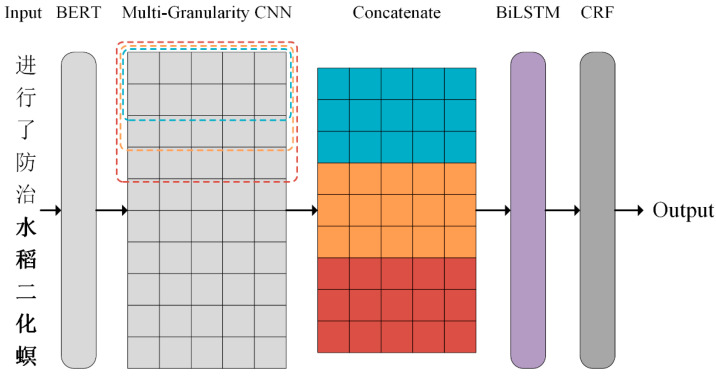
Overall model architecture (The Chinese in the figure is the original text input, which means “Prevention and control of rice stem borer”).

The overall architecture of the model is shown in Figure 3.

As the input layer of the model, BERT [25] is responsible for converting the input Chinese text into rich semantic representation. Through its pre-trained context representation, BERT can capture the syntax and semantic information in the text, providing a strong language foundation for the subsequent NER tasks. Inspired by the biological vision system, CNNs with different sizes of convolution kernel are used to extract multi-granularity features, so that the model can better learn the information of nested entities. A BiLSTM is used to capture bidirectional dependencies in text and model sequences. A CRF is used to model the dependency relationship between tags. It can efficiently calculate the optimal tag sequence by decoding with dynamic programming algorithm, so as to improve the accuracy of NER.

### 3.2. BERT Encoder

BERT is a pre-training language model based on transformer architecture, which aims to generate high-quality language representation through unsupervised learning.

The core architecture of BERT is a transformer [26], which encodes the input sequence through a Multi-Head Self-Attention mechanism and a Feed-Forward Neural Network. BERT uses a Masked Language Model (MLM) task to randomly mask some words in the input sequence and then predicts these masked words. This bi-directional modeling method can make full use of context information to generate high-quality language representation. BERT learns the universal language representation through pre-training on large-scale corpora. The pre-training task includes an MLM and Next Sentence Prediction, which can capture the syntax and semantic information of the language. BERT adapts to downstream tasks through fine-tuning, such as text classification, NER, etc. The fine-tuning process is simple and efficient and can quickly adapt to different task requirements.

BERT performs well in natural language processing tasks, especially in text classification, NER, and other tasks. The pre-training representation of BERT can capture rich semantic information and provide a strong language foundation for downstream tasks. For example, in the task of Chinese NER, BERT can better understand the entity information in the text through the pre-trained context representation, so as to improve the accuracy of NER.

### 3.3. Multi-Granularity CNN

A CNN can extract n-gram information from text [27]. Its core idea is to capture local features. For text, local features are sliding windows composed of several words, similar to n-gram, as shown in Figure 3. The advantage of convolutional neural networks is that they can automatically combine and filter n-gram features to obtain semantic information at different levels of abstraction.

Inspired by the biological vision system, a set of CNNs with different convolution kernel sizes is used to extract multi-granularity features to better capture nested entity information. Specifically, directly following the BERT embedding layer, we deploy a set of parallel CNNs with kernel sizes designed to model different linguistic scopes: small kernels detect character-level patterns and suffixes indicative of entity types, while larger kernels capture key local context words. The concatenated multi-granular features are then fed into the BiLSTM, providing it with a richer, pre-processed signal for sequence modeling. This targeted architectural choice enhances the model’s ability to discern nested entities. Record the text vector encoded by BERT as $H_{0}\in R^{N\times d_{e}}$, where N is the sequence length and $d_{e}=7$68 is the BERT vector dimension. Record multiple CNNs used for multi-granularity feature extraction as CNN_1_, CNN_2_, …, CNN_C_, where C is the number of CNNs used. Then, the extracted multi-granularity information is as follows:

(1) $$H_{i}={CNN}_{i}\left(H_{0}\right)\in R^{N\times d_{c}},i\in [1,C],$$

where $d_{c}$ is the output dimension of the CNN.

Then, connect the output of each CNN to obtain the output of the multi-granularity CNN:

(2) $$H_{m}=H_{1}⨁H_{1}⨁\cdots ⨁H_{C}\in R^{N\times {Cd}_{c}},$$

By fusing the outputs of different convolution layers, the model can adaptively combine short-range word order features with long-range dependence features and enhance the representation ability of composite nested entities. The small-scale convolution kernel focuses on the key components inside the entity, while the large-scale convolution kernel captures the modification or subordination between entities and accurately locates the nested boundary.

### 3.4. BiLSTM

The basic unit of LSTM is a memory cell. Each memory cell contains three gates, the forgetting gate, the input gate and the output gate [28]. The functions of these doors are as follows.

The forgetting gate determines which information in the memory unit needs to be forgotten. The calculation formula is as follows:

(3) $$f_{t}=\sigma (W_{f}\cdot [h_{t-1},x_{t}]+b_{f}),$$

where $f_{t}$ is the output of the forgetting gate, σ is the sigmoid activation function, $W_{f}$ is the weight matrix, $b_{f}$ is the offset term, $h_{t-1}$ is the hidden state of the previous time, and $x_{t}$ is the input of the current time.

The input gate determines which new information needs to be written into the memory unit, including two parts: the active value and candidate value of the input gate. The calculation formula is as follows:

(4) $$i_{t}=\sigma (W_{i}\cdot [h_{t-1},x_{t}]+b_{i}),$$

(5) $${\overset{\sim }{c}}_{t}=tanh(W_{c}\cdot [h_{t-1},x_{t}]+b_{c}),$$

where $i_{t}$ is the activation value of the input gate, ${\overset{\sim }{c}}_{t}$ is the candidate value, and tanh is the hyperbolic tangent activation function.

The output gate determines which information in the memory unit needs to be output. The calculation formula is as follows:

(6) $$o_{t}=\sigma (W_{o}\cdot [h_{t-1},x_{t}]+b_{o}),$$

(7) $$h_{t}=o_{t}\cdot tanh(c_{t}),$$

where $o_{t}$ is the activation value of the output gate and $h_{t}$ is the hidden state at the current time.

The status update formula of the memory unit is as follows:

(8) $$c_{t}=f_{t}⊙c_{t-1}+i_{t}⊙{\overset{\sim }{c}}_{t},$$

Through the above gating mechanism, LSTM can effectively control the flow of information, so as to solve the problem of gradient disappearance in long series of data.

In order to enable the model to learn the forward and backward information in the sequence, the forward LSTM and the reverse LSTM are combined to obtain the output of the BiLSTM:

(9) $$H_{l}=\vec{LSTM}(H_{m})⨁\overset{\leftarrow }{LSTM}(H_{m})\in R^{N\times {2d}_{l}},$$

where is the LSTM hidden layer dimension.

### 3.5. Conditional Random Fields

CRF is a conditional probability model used to model the conditional probability distribution between tag sequence Y and observation sequence X [29,30]. CRF is defined as follows:

(10) $$P(Y|X)=\frac{1}{Z(X)}\exp(\sum_{k}\lambda_{k}f_{k}\left(Y,X\right)),$$

where Z(X) is the normalization factor, $\lambda_{k}$ is the weight of the feature function $f_{k}$, and $f_{k}\left(Y,X\right)$ is the feature function, which is used to measure the matching degree between the tag sequence Y and the observation sequence X, and to model the dependency between tags in the sequence annotation task, so as to improve the accuracy of annotation.

## 4. Results

### 4.1. Experimental Environment and Parameter Setting

In the experiments, the entire model was implemented by PyTorch 2.1.0 (https://pytorch.org/ (accessed on 5 August 2025)) in a Windows 11 environment, and chinese-bert-wwm-ext BERT pre-trained weights (https://huggingface.co/hfl/chinese-bert-wwm-ext (accessed on 5 August 2025)) were used in the encoder, fine-tuned as the model was trained. We trained our model on a single NVIDIA GeForce RTX 4090 GPU. The hyper-parameter settings are shown in Table 2:

Strict evaluation metrics are used, i.e., entities are identified as correctly predicted when both entity boundaries and entity types are correct, otherwise, they are incorrectly predicted. Performance is evaluated using precision (P), recall (R), and F1 scores (F).

### 4.2. Comparison Results

We reproduced the mainstream NER method and a variety of recent advanced methods as the baseline and compared them with our proposed model. The results are shown in Table 3.

As shown in Table 3, our proposed model is superior to all baseline models in terms of accuracy (P), recall (R), and F1 value (f), showing its effectiveness in the task of NER. Specifically, our model achieved 90.86% accuracy, 92.64% recall, and 91.74% F1 value. Compared with the best performance baseline model BERT-Biaffine (P: 89.89%, R: 92.40%, F: 91.13%), our model improved the F1 value by 0.61 percentage points. This improvement is mainly due to the significant improvement in precision (+0.97 percentage points), while the recall rate also maintained a high level (+0.24 percentage points), indicating that our method achieves a better balance between accurately identifying entities and finding all entities as much as possible. The multi-granularity feature is extracted by the CNNs with different convolution kernel sizes, which optimizes the determination of entity boundary, thus significantly improving the recognition precision while maintaining a high recall rate.

### 4.3. Influence of Multi-Granularity CNNs

**Figure 4:**
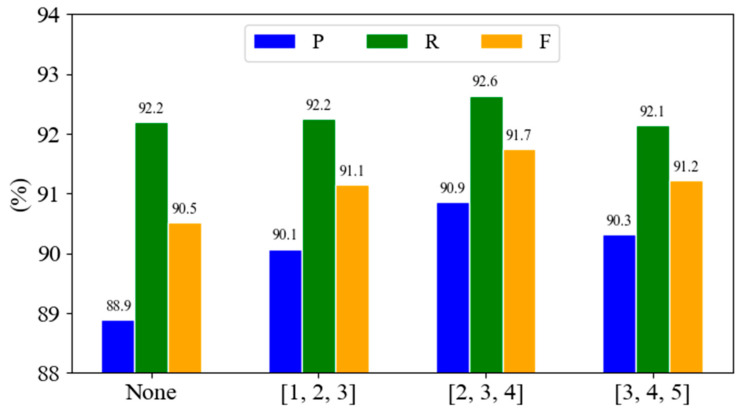
Ablation results of triple convolution kernel combination.

In order to further illustrate the effect of multi-granularity CNNs, experiments without CNNs and using different combinations of CNN convolution kernels were constructed. A CNN was not used and convolution kernels with sizes of [1, 2, 3], [2, 3, 4], [3, 4, 5] were used. The experimental results are shown in Figure 4.

As shown in Figure 4, the introduction of a multi-granularity CNN module significantly improves the performance of the model. Compared with the 90.5% F1 score obtained without a CNN, all CNN combinations bring positive gain. The best combination [2, 3, 4] achieved 91.7% F1 score, an increase of 1.2 percentage points. When the convolution kernel combination increases to [3, 4, 5], the F1 value decreases to 91.2%, which indicates that too large convolution kernels are not conducive to the performance improvement of the model.

**Figure 5:**
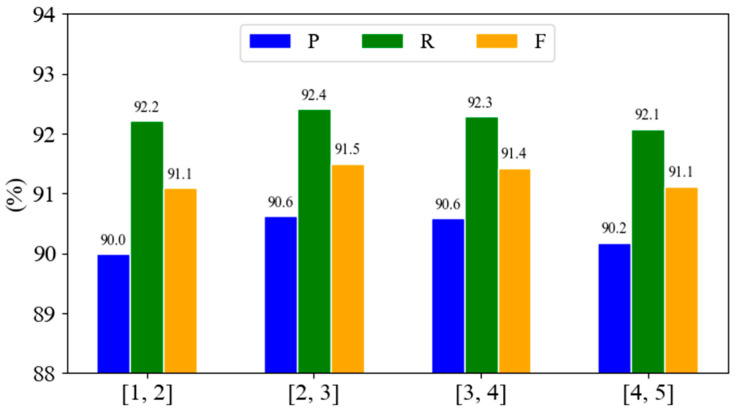
Ablation results of double convolution kernel combination.

The convolution kernel combinations with sizes of [1, 2], [2, 3], [3, 4], [4, 5] were used. The experimental results are shown in Figure 5.

The double convolution kernel experiment in Figure 5 further verified that the [2, 3] combination performed best, achieving an F1 score of 91.5%, which can well balance local features and context dependence. When using a [4, 5] large-scale convolution kernel, the F1 score was 91.1%, indicating that excessive dependence on long-distance features will introduce noise and reduce performance. When using a [1, 2] small-scale convolution kernel, the F1 score was also 91.1%, indicating that insufficient capture of long-distance features will also reduce performance. The small-scale convolution kernel ([1, 2]) effectively identifies the basic entity units, while the mesoscale kernel captures the modification relationship to realize the hierarchical decoding of the nested structure.

**Figure 6:**
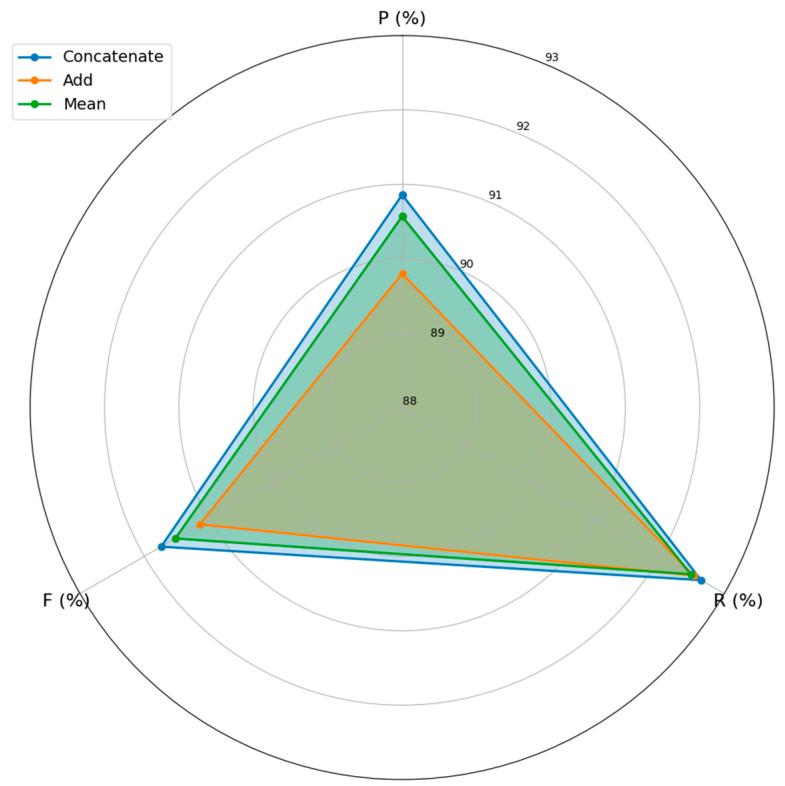
Performance of different fusion strategies.

In order to verify the impact of different fusion strategies on performance, a comparative experiment using additive and meaning strategy and concatenating strategy was constructed. The experimental results are shown in Figure 6.

As can be seen from Figure 6, the three fusion strategies can effectively extract features, and the concatenating strategy is the best. The effect of the measuring strategy is similar, but the effect of the additive strategy is relatively poor.

In summary, reasonable selection of convolution kernel size and the concatenating strategy are helpful for the modeling of text features and then the improvement of performance.

### 4.4. Influence of CNN Hidden Layer Dimension

The hidden layer dimension in a CNN is a pivotal hyperparameter that governs the richness of the feature space at each layer and the model’s overall capacity. A higher dimension enables the network to capture more diverse and finer-grained local patterns, thereby enhancing its ability to model complex, non-linear relationships—a capability essential for handling the ambiguity and context-dependency inherent in natural language. However, this involves a critical trade-off. Increasing the dimension boosts model complexity but also escalates the risk of overfitting (especially with limited data) and computational cost.

Therefore, experiments on different CNN hidden layer dimensions are constructed to explore the best dimension selection, and the results are shown in Figure 5.

**Figure 7:**
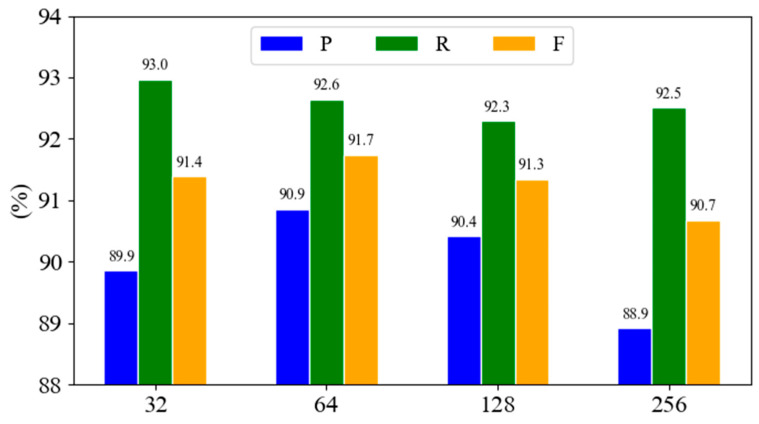
Performance of different CNN hidden layer dimensions.

From Figure 7, it can be seen that the hidden layer dimension of a CNN has a significant impact on the model’s performance. When the hidden layer dimension is 64, the model achieves the best performance (P: 92.6, R: 92.3, F: 92.5), indicating that the dimension achieves a good balance between capturing local features and global semantics. When the dimension further increases to 256, the three indicators decrease significantly, suggesting that the model may have overfitting due to too many parameters. In contrast, when the dimension is 32, the model performance is better than the larger dimension, but still lower than the middle dimension (64 and 128), indicating that a certain diversity of features is the key to improving the model’s expression ability, but too high a dimension will damage the generalization ability. To sum up, the choice of hidden layer dimension needs to be balanced between model capacity and generalization performance. This experiment shows that the dimension of 64 is a better choice in this task scenario.

### 4.5. Categorical Performances

To comprehensively evaluate the performance of our proposed model beyond the overall metrics, we present a detailed per-category analysis. Table 4 delineates the performance for each specific entity type in the test set.

This breakdown is crucial for identifying the model’s strengths and weaknesses across different entity types, which may be obscured by the macro-averaged scores. The results presented in Table 4 reveal significant variations in model performance across different entity categories, indicating that its recognition capability is highly influenced by the semantic and contextual features specific to each entity type. The model demonstrates particularly strong performance on core entities such as Disease, Pests, Crop and Cultivar, with F1 scores of 97.53%, 95.10%, 94.18%, and 90.29%. These entities constitute the central focus within the domain of rice diseases and insect pests, typically characterized by standardized nomenclature and contextually distinctive vocabulary, thereby forming the practical basis of the entire system.

The model demonstrates moderate yet slightly inferior performance on entities such as Drug, Pathogens, Part, Fertilizer, and Period. Although these entities are also closely associated with agricultural production practices, their names tend to exhibit greater variability or ambiguity. For instance, pesticide names may incorporate complex chemical nomenclature, trade names, and abbreviations, while temporal expressions related to periods might be confused with general time references.

Notably, the model performs relatively poorly on entities belonging to biosystematic categories and organizations. Biosystematic entities often involve specialized Latin scientific names and hierarchical taxonomic terms, which occur infrequently in texts and across diverse contexts, resulting in insufficient model learning. The identification of organization names remains challenging due to the virtually unlimited variability of their compositions and frequent overlaps with common nouns.

In summary, the performance analysis presented in this section confirms that our model is highly effective in recognizing core entities with well-defined semantics and consistent contextual patterns within the domain of rice diseases and pests. However, it encounters difficulties when processing entity types that are highly specialized, sparse, or semantically ambiguous. Furthermore, the low performance on the Other category suggests that the current entity classification schema could be refined by introducing more semantically consistent subcategories, thereby improving the overall recognition capability of the model.

### 4.6. Ablation Study

The model with different components eliminated is tested on the part of nested entities in the test set and the whole test set after training to verify our key improvements. The results are shown in Table 5.

As illustrated in Table 5, the results of the ablation study clearly validate the effectiveness of the key components in our model, particularly highlighting the significant contribution of the multi-granularity CNN module to nested entity recognition.

On the subset of nested entities, removing the multi-granularity CNN module led to a decline in the F1 score from 90.33% to 87.21%, a reduction of 3.12 percentage points. In contrast, the F1 score on the entire test set decreased by 1.23 percentage points. This discrepancy indicates that the performance gain offered by the multi-granularity CNN is especially pronounced for nested entity recognition.

Nested entities often exhibit complex structures, ambiguous boundaries, and encompass semantic information at different hierarchical levels. Conventional single-scale feature extractors struggle to adequately capture the internal structure and contextual dependencies of such entities. The multi-granularity CNN addresses this limitation by employing convolutional kernels of varying sizes, enabling the simultaneous extraction of local detailed features and broader contextual features. This capability is crucial for recognizing internal sub-structures within entities and the multi-level relationships between an entity and its surrounding context. For instance, smaller kernels aid in identifying short phrases or terms inside an entity, while larger kernels help comprehend the broader context in which the entity appears, which is paramount for disambiguating the boundaries and categories of nested entities.

Furthermore, BERT contributes most critically, as replacing it with Word2Vec precipitates a sharp F1 score decline of over 9 percentage points, underscoring the foundational role of its contextualized embeddings in disambiguating entities. The BiLSTM layer is of secondary yet significant importance, demonstrating the necessity of modeling long-range sequential dependencies for accurate boundary detection. The multi-granularity CNN specifically enhances the recognition of nested entities, while this effect cannot be achieved without using a CNN or using simple CNNs. Each component addresses a distinct level of feature abstraction, and their combination is essential for the model’s strong performance, particularly in handling complex nested structures.

## 5. Conclusions

To address the challenge of nested named entity recognition in texts related to rice diseases and pests, this study constructs a large-scale, high-quality dataset (comprising 15,000 sentences and 56,657 entities across 13 fine-grained entity types) for the rice bio-threat domain. Inspired by biological vision mechanisms, a multi-granularity feature fusion model is proposed. This model achieves hierarchical decoding of nested structures through parallel multi-scale convolutional kernels, attaining an optimal F1 score of 91.74% on the constructed dataset. Experimental results demonstrate that the multi-granularity CNN effectively enhances the detection of nested boundaries: small kernels enable precise localization of elementary entities, while medium-sized kernels capture modifying relationships. Future work will focus on developing a cross-modal joint recognition model that integrates image features of diseases and pests to further improve recognition performance, and expanding the data sources to include social media, farm monitoring reports, and real-time sensor data to enhance the model’s robustness.

## Figures and Tables

**Table 1:** Details of entities categories.

Number	Tags (EN)	Abbr.	Quantity
1	Biosystematic	BIS	283
2	Company	COM	303
3	Crop	CRO	14,936
4	Cultivar	CUL	4443
5	Disease	DIS	9058
6	Drug	DRUG	6250
7	Fertilizer	FER	327
8	Organization	ORG	1216
9	Other	OTH	456
10	Pathogens	PAOG	1024
11	Part	PART	2595
12	Period	PER	4361
13	Pests	PET	11,405

**Table 2:** Hyper-parameter settings.

Hyper-Parameters	Value
loss function	cross entropy
optimizer	Adam
learning rate	0.001
CNN convolution kernel	[2, 3, 4]
CNN hidden layer dimension $(d_{c}$)	64
LSTM hidden layer dimension $(d_{l}$)	128
sequence length (N)	100
dropout	0.1
batch size	32

**Table 3:** Comparison between baseline model and proposed model (%).

Method	P	R	F
BERT-BiLSTM-CRF	88.89	92.19	90.51
BERT-BiGRU-CRF	88.97	90.14	89.55
W^2^NER [31]	90.01	92.12	91.04
DiffusionNER [32]	90.12	91.62	90.84
DiFiNet [33]	90.34	91.59	90.96
BERT-Biaffine [17]	89.89	92.40	91.13
**Ours**	**90.86**	**92.64**	**91.74**

**Table 4:** Categorical performances (%).

Entities Type	P	R	F
Biosystematic	71.43	86.21	78.13
Company	78.79	81.25	80.00
Crop	93.88	94.49	94.18
Cultivar	87.20	93.60	90.29
Disease	97.36	97.70	97.53
Drug	89.56	89.73	89.65
Fertilizer	80.95	87.18	83.95
Organization	65.96	82.30	73.23
Other	55.00	42.31	47.83
Pathogens	85.32	92.08	88.57
Part	87.18	87.93	87.55
Period	81.45	81.06	81.25
Pests	94.27	95.94	95.10

**Table 5:** Results of ablation study (%).

Components	The Part of Nested Entities			Whole Test Set		
	P	R	F	P	R	F
Origin	89.12	91.57	90.33	90.86	92.64	91.74
Replace multi-granularity CNN with simple CNN	87.19	88.69	87.93	89.90	91.35	90.64
Without multi-granularity CNN	86.20	88.24	87.21	88.89	92.19	90.51
Replace BERT with Word2Vec	81.29	80.49	80.89	82.17	81.21	81.69
Without BiLSTM	83.97	88.18	86.02	86.86	88.67	87.76
Replace BiLSTM with simple RNN	86.43	88.26	87.34	88.18	89.18	88.68

## Data Availability

The data presented in this study are available on request from the corresponding author. The data are not publicly available due to privacy.
